# The Impact of the COVID-19 Pandemic on Latinx College Students’ Mental Health: Exploring the Role of Sleep, Social Support, and Attitudinal *Familismo* Support

**DOI:** 10.3390/clockssleep8030049

**Published:** 2026-08-21

**Authors:** Mayra S. Ramos, Pablo Soto, Chelsea Derlan Williams, Daniella Olivares, Alisandra Macias, Jennifer M. Rohan, Gabriela León-Pérez, Kevin Allison, Natalie Dautovich, Joseph M. Dzierzewski, Rosalie Corona

**Affiliations:** 1 Department of Psychiatry and Behavioral Health, Ann & Robert H. Lurie Children’s Hospital of Chicago, Chicago, IL 60611, USA; 2 College of Humanities and Sciences, Department of Psychology, Virginia Commonwealth University, Richmond, VA 23284, USAolivaresd@vcu.edu (D.O.); racorona@vcu.edu (R.C.); 3 Indiana University School of Medicine and Riley Children’s Health, Indianapolis, IN 46202, USA; jrohan@iuhealth.org; 4 College of Humanities and Sciences, Department of Sociology, Virginia Commonwealth University, Richmond, VA 23284, USA; gleonperez@vcu.edu; 5 National Sleep Foundation, Washington, DC 20036, USA

**Keywords:** COVID-19, discrimination, mental health, *familismo*, sleep

## Abstract

College students experienced various disruptions to their daily life and changes to their physical and mental health because of the COVID-19 pandemic. Much remains unknown about protective factors that could buffer the negative impact of the COVID-19 pandemic on Latinx college students’ mental health. Cross-sectional survey data collected during the pandemic was used to explore whether global sleep quality mediated associations between COVID-19 factors (i.e., negative experiences and racial/ethnic bias) and mental health outcomes (i.e., depressive and anxiety symptoms), and whether perceived social support and attitudinal *familismo* support served as moderators of this association. The current study included 490 Latinx college students (*M*_age_ = 19.1, 72.9% female). The mediation model had acceptable fit: χ^2^ (*df* = 4) = 11.243, *p* = 0.024; RMSEA = 0.06; CFI = 0.99, SRMR = 0.01. Mediation results indicated that negative COVID-19 experiences (*β* = 0.12, 95% CI [0.09, 0.16], *p* = 0.001) and COVID-19 racial/ethnic bias (*β* = 0.04, 95% CI [0.01, 0.07], *p* = 0.023) were associated with worse global sleep quality, and, in turn, more severe mental health outcomes. There were no significant moderation effects. Implications for possible interventions to support Latinx college students’ mental health, and future directions are discussed.

## 1. Introduction

The COVID-19 pandemic negatively impacted college students’ mental and physical health. In several countries, college students reported poorer mental health [1,2] and worse health-related quality of life [3], compared to non-students, during the pandemic. Latinx college students reported increased anxiety related to the economic strain (e.g., loss of employment, inability to pay bills) caused by the pandemic to themselves, family members, and friends) [4]. Latinx first year college students were also more likely than their White peers to report difficulties with distance learning [5], adding stress related to their academic progress. Latinx college students experienced changes in their education, living situation, and academic performance while navigating the impact of the pandemic on their communities and families [6]. The stressors associated with the pandemic had a negative impact on their mental health. Indeed, Latinx college students reported an increase in mental health problems (e.g., symptoms of anxiety, depression, stress) during and after the pandemic [7,8].

In addition to dealing with the impact of COVID-19 on their schooling, families, and communities, Latinx college students were also faced with increased racial/ethnic bias (e.g., discrimination, distress associated with victimization experiences) because of anti-immigrant rhetoric during and after the pandemic [9,10,11]. Experiences from this “double pandemic” left them vulnerable to worse mental health outcomes [12]. For instance, 51% of Latinx college students reported vicarious discrimination (i.e., witnessing discriminatory behavior experienced by others) because of heightened anti-immigrant sentiments during the COVID-19 pandemic [9]. After adjusting for covariates (e.g., age, gender), direct and indirect discrimination were significantly associated with Latinx college students’ psychological distress, financial hardships due to the pandemic, and loss of a loved one due to COVID-19 [9]. Others have also found that COVID-19 racial/ethnic bias was associated with poorer mental health among Latinx college students [13,14]. Together, these findings highlight the importance of identifying factors that explain the impact of the pandemic on the well-being of Latinx college students. Of possible mediating factors, global sleep quality may indirectly explain the mental health changes associated with the COVID-19 pandemic.

### 1.1. COVID-19 and Global Sleep Quality: A Potential Mediator

The onset of the pandemic and the continued stressors that occurred altered sleep quality and routines [15]. A systematic review and meta-analysis by Jahrami et al. [16] reported that the global pooled prevalence rate of sleep problems among all populations across various nations was nearly 36%. For instance, Benham [17] found that Latinx college students reported later sleep and wake times, greater sleep duration, longer times to fall asleep, and less sleep efficiency during the pandemic compared to pre-pandemic patterns. Further, Latinx college students who reported that the pandemic had worsened their sleep health reported greater daytime tiredness, fatigue, and sleepiness [18]. Changes in sleep patterns and quality are concerning because they are associated with poorer mental health outcomes in the general population (see meta-analysis by Scott et al. [19]) and for college students [20,21]. For example, sleeping less and daytime tiredness/fatigue/sleepiness were associated with a higher prevalence of psychological distress and worse mental health status among Latinx college students [18].

Further, experiences of racial/ethnic bias are negatively associated with Latinx college students’ sleep quality. Using a weekly diary approach, Davenport et al. [22] found that on pre-pandemic weeks in which African American and Latinx college students reported more experiences of racial microaggressions, they also reported poorer sleep and a harder time falling asleep. Similarly, Franklin et al. [23] found a significant association between experiencing microaggressions and reports of inability to sleep and sleeping less among Latinx college students. Research during and after the pandemic suggests that Latinx adults’ experiences of COVID-19-related bias were not associated with changes in their sleep quality [24,25] nor in sleep duration [25].

Finally, pre-pandemic findings are mixed as to whether sleep explains the relationship between racial/ethnic discrimination and Latinx adults’ negative mental health outcomes, with some studies finding significant mediation [26,27] among Latinx college students and adults, and others finding no mediation effects [28]. Williams and colleagues [29] found that the association between stress associated with housing instability at the beginning of the pandemic and college students’ mental health and physical health outcomes a year later was mediated by sleep dissatisfaction during the pandemic. The current study builds on this emerging literature by exploring whether global sleep quality, composed of multiple sleep characteristics, serves as a mediator between COVID-19 factors (i.e., COVID-19 experiences and COVID-19 racial/ethnic bias) and Latinx college students’ mental health. In addition, we aim to identify culturally relevant moderators of this mediated model.

### 1.2. Culturally Relevant Moderators: Behavioral and Attitudinal Familismo Support

Among Latinxs, an important cultural value is *familismo*, which is comprised of attitudinal and behavioral components [30]. Attitudinal *familismo* support are beliefs and attitudes that emphasize the warmth and closeness of supportive familial relationships within Latinx families [31]. Behavioral *familismo* support is the actions and behaviors that stem from these beliefs and attitudes [32]. Much of the literature on *familismo* focuses primarily on the attitudinal component, which has produced mixed findings. To help clarify findings, it is recommended that researchers clearly indicate which aspect of *familismo* is being investigated while also emphasizing the need for more work focused on behavioral *familismo* [30,31]. In the current study, we include a measure of attitudinal *familismo* support and a proxy measure of behavioral familismo (i.e., social support).

#### 1.2.1. Attitudinal Familismo Support

The protective role of attitudinal *familismo* support on mental health has been studied among Latinx adolescent and emerging adult populations, including college students [32]. For example, Corona et al. [33] found that attitudinal *familismo* referent (e.g., behaving in ways consistent with family expectations) moderated the association between perceived discrimination and Latinx college students’ symptoms of anxiety. Likewise, a study by Killoren et al. [34] reported that attitudinal *familismo* support was associated with fewer symptoms of depression for Latina college students. Familism values may also protect Latinxs sleep health. For example, Sasser and others [35] found that attitudinal familism obligation moderated the association between average time spent with siblings and sleep efficiency such that greater attitudinal familism obligation strengthened the negative association between sibling connection and sleep efficiency. While some studies have found that attitudinal *familismo* may serve as a risk factor for psychosocial adjustment and sleep health, a recent meta-analysis found that attitudinal *familismo* functions more as a ‘promotive’ factor than risk factor across educational, family relationship, and mental health outcomes [32]. These pre-pandemic findings suggest that attitudinal *familismo* support may buffer the association between pandemic experiences and Latinx college students’ global sleep quality, which in turn may be associated with their mental health.

#### 1.2.2. Social Support

While few measures of behavioral *familismo* currently exist (see Nicasio et al. [36] for an exception), constructs such as social support may be considered proxy measures of behavioral *familismo*. To support this idea, Latinx college students identified seeking support from family and peers as an adaptive coping strategy pre-pandemic [30,37] and during the pandemic [8], suggesting that social support from one’s family is an important aspect of *familismo*. Studies have found social support to be a protective factor that reduces the association between COVID-19 worry or stress and college students’ psychological health, alcohol use, and sleep quality [38,39]. In contrast, Serpas and Ignacio [14] did not find social support was a significant moderator between COVID-19 anxiety, fear, and racial/ethnic bias and symptoms of depression, anxiety, and stress for Latinx college students. These mixed findings warrant further attention.

### 1.3. Current Study

The current study uses original survey data collected during the pandemic to assess whether global sleep quality mediated the relations among negative COVID-19 experiences and racial/ethnic bias predicting mental health outcomes (i.e., symptoms of depression and anxiety). We hypothesized that more negative COVID-19 experiences (e.g., negative impact of the pandemic on students) and racial/ethnic bias would be associated with poorer global sleep quality, which would, in turn, be associated with greater depressive and anxiety symptoms. In addition, we tested two moderated mediation models to explore attitudinal *familismo* support and social support as moderators. We hypothesized that attitudinal *familismo* support and social support would significantly moderate the associations between COVID-19 factors predicting worse mental health symptoms, such that this risk pathway would be significantly weakened for those with greater attitudinal *familismo* support and social support.

## 2. Results

Bivariate correlations, means and standard deviations for the study measures are provided in Table 1. Possible covariates such as age, immigrant generation, months since the COVID-19 lockdown, and Latinx racial/ethnic identity were not significantly associated with the ‘mental health outcomes’ latent variable. As such, they were not retained as covariates. However, gender identity was included in the analyses as a covariate given it was significantly associated with mental health outcomes. Nearly all students (91%) reported that they or someone they knew had been exposed to the COVID-19 virus over the past two weeks. Approximately 42% of students reported that they ‘somewhat agreed’ with the belief that the country had become more dangerous for people of their racial/ethnic group because of fear of the coronavirus. Similarly, most students agreed that people of their race/ethnicity were more likely to lose their job or would not receive coronavirus healthcare as good as the care received by other groups (e.g., 44% and 46%, respectively). In addition, 73.7% of the students reported a global sleep quality score of 5 or higher [40]. Nearly one-third (31.4%) of students endorsed mild depressive symptoms, 25.7% endorsed minimal depressive symptoms, 21.6% endorsed moderate depressive symptoms, 12.7% endorsed moderately severe depressive symptoms, and 8.6% severe depressive symptoms, on the PHQ-9. On the GAD-7, 33.3% of students reported minimal anxiety, 30% reported mild anxiety, 21.2% reported moderate anxiety, and 15.5% reported severe anxiety.

First, we ran the measurement model, which had acceptable fit: χ^2^ (*df* = 3) = 11.198, *p* < 0.011; RMSEA = 0.08; CFI = 0.99, SRMR = 0.01. The manifest indicators, depressive symptoms and anxiety symptoms, for the mental health latent variable loaded highly (standardized beta weighs > 0.88, *p* < 0.001).

### 2.1. Mediation Model

The mediation model had acceptable fit: χ^2^ (*df* = 4) = 11.243, *p* = 0.024; RMSEA = 0.06; CFI = 0.99, SRMR = 0.01. Results indicated that more negative COVID-19 experiences and COVID racial/ethnic bias were associated with poorer global sleep quality and, in turn, worse mental health outcomes (i.e., greater symptoms of anxiety and depression), and both mediation pathways were statistically significant. Specifically, the indirect effect of COVID-19 experiences on mental health outcomes through global sleep quality was significant (*β* = 0.12, 95% CI [0.09, 0.16], *p* = 0.001) and the indirect effect of COVID-19 racial/ethnic bias and mental health outcomes through global sleep quality (*β* = 0.04, 95% CI [0.01, 0.07], *p* = 0.023) was significant (see Table 2 for analysis summary). Additionally, four direct effect paths were significant, such that more negative COVID-19 experiences, COVID-19 racial/ethnic bias, poor global sleep quality, and identifying as a female, were directly associated with worse depressive and anxiety symptoms.

### 2.2. Moderated Mediation Models: Attitudinal Familismo Support and Social Support as Moderators

#### 2.2.1. Attitudinal Familismo Support as the Moderator

**Figure 1:**
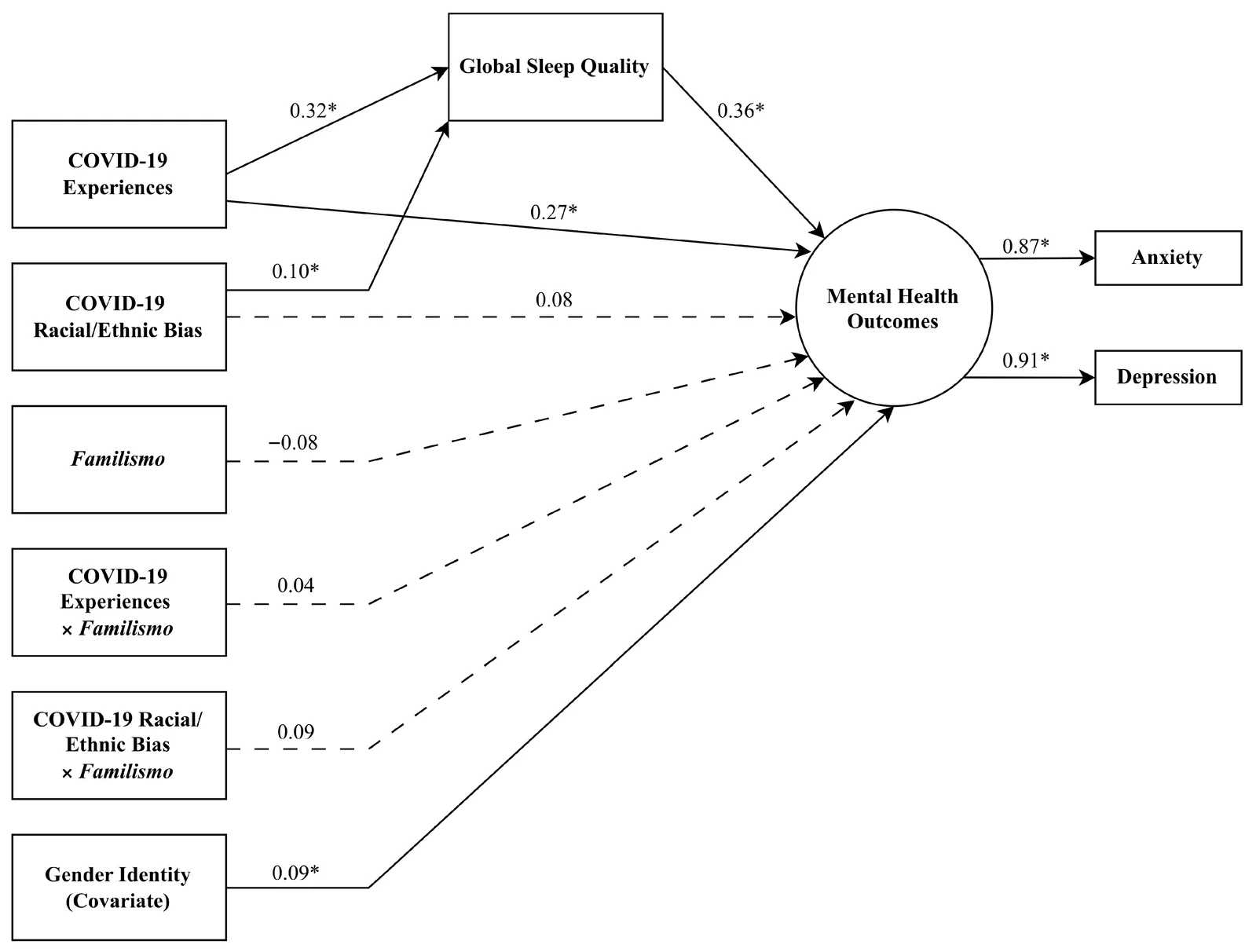
Moderated mediation model of associations between COVID-19 experiences and bias, with mental health via global sleep quality moderated by attitudinal *familismo* support. Note: Attitudinal *familismo* support = *familismo*. Values on paths are standardized *β* coefficients. Solid paths indicate statistically significant effects (* *p* < 0.05), whereas dashed paths indicate non-significant effects.

The first moderated mediation model assessed whether Latinx college students’ attitudinal *familismo* support moderated the relation between COVID-19 experiences and COVID-19 racial/ethnic bias predicting mental health outcomes via global sleep quality. This model had acceptable fit: χ^2^ (*df* = 14) = 54.48, *p* < 0.001; RMSEA = 0.08; CFI = 0.95, SRMR = 0.04 (Figure 1). Findings from this model indicated that there were no significant moderation effects of attitudinal familism support. In addition, global sleep quality mediated the association between COVID-19 experiences and mental health outcomes (95% CI = 0.44, 0.80) and between COVID-19 racial/ethnic bias and mental health outcomes (95% CI = 0.06, 0.33). In addition, one direct effect was also significant such that more COVID-19 experiences were associated with worse mental health (see Figure 1 and Table 3 for direct effect statistics). Consistent with the other analyses, female participants were more likely to report more severe mental health symptoms than males.

#### 2.2.2. Social Support as the Moderator

**Figure 2:**
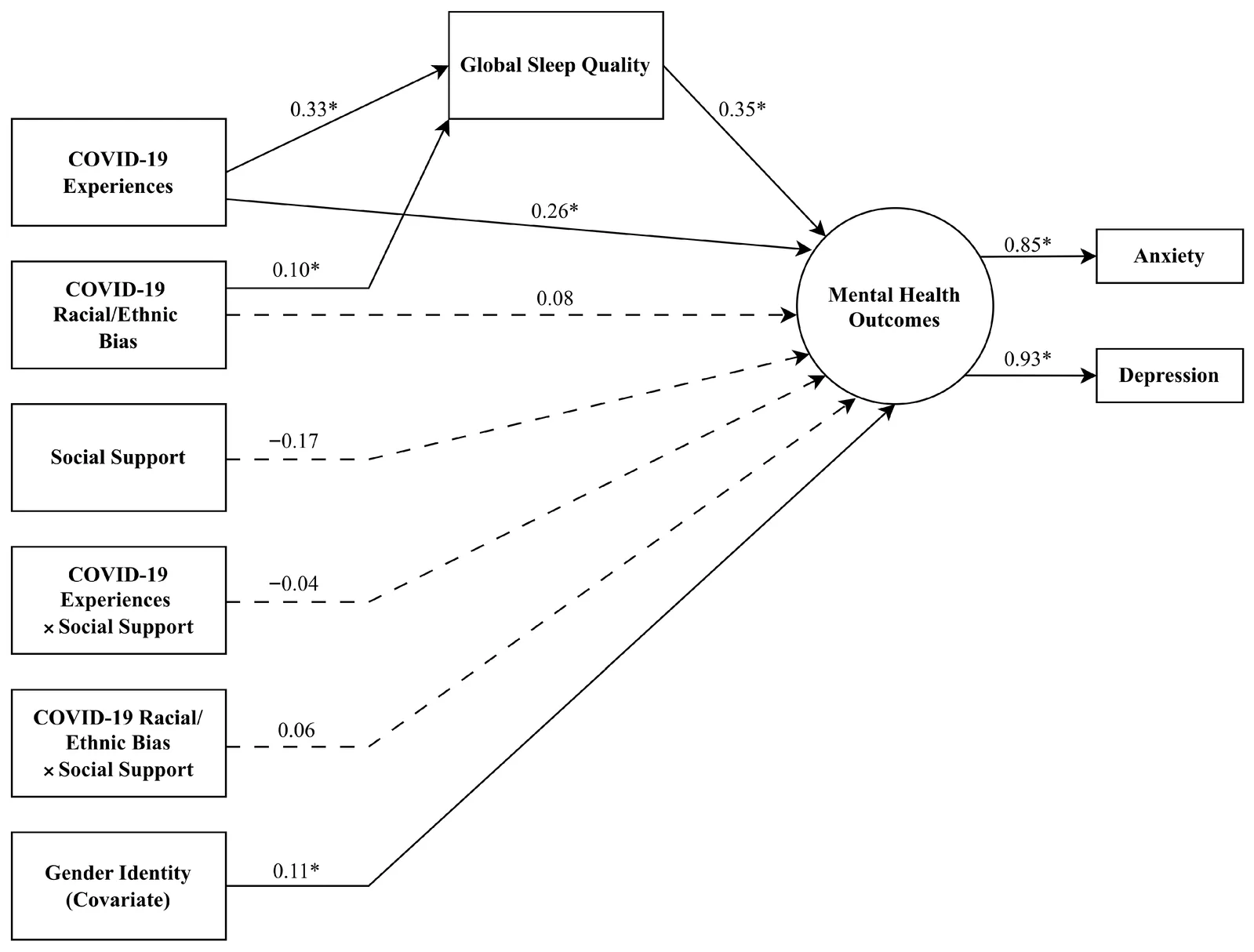
Moderated mediation model. Associations between COVID-19 experiences and bias with mental health via global sleep quality moderated by social support. Note: Values on paths are standardized *β* coefficients. Solid paths indicate statistically significant effects (* *p* < 0.05), whereas dashed paths indicate non-significant effects.

**Figure 3:**
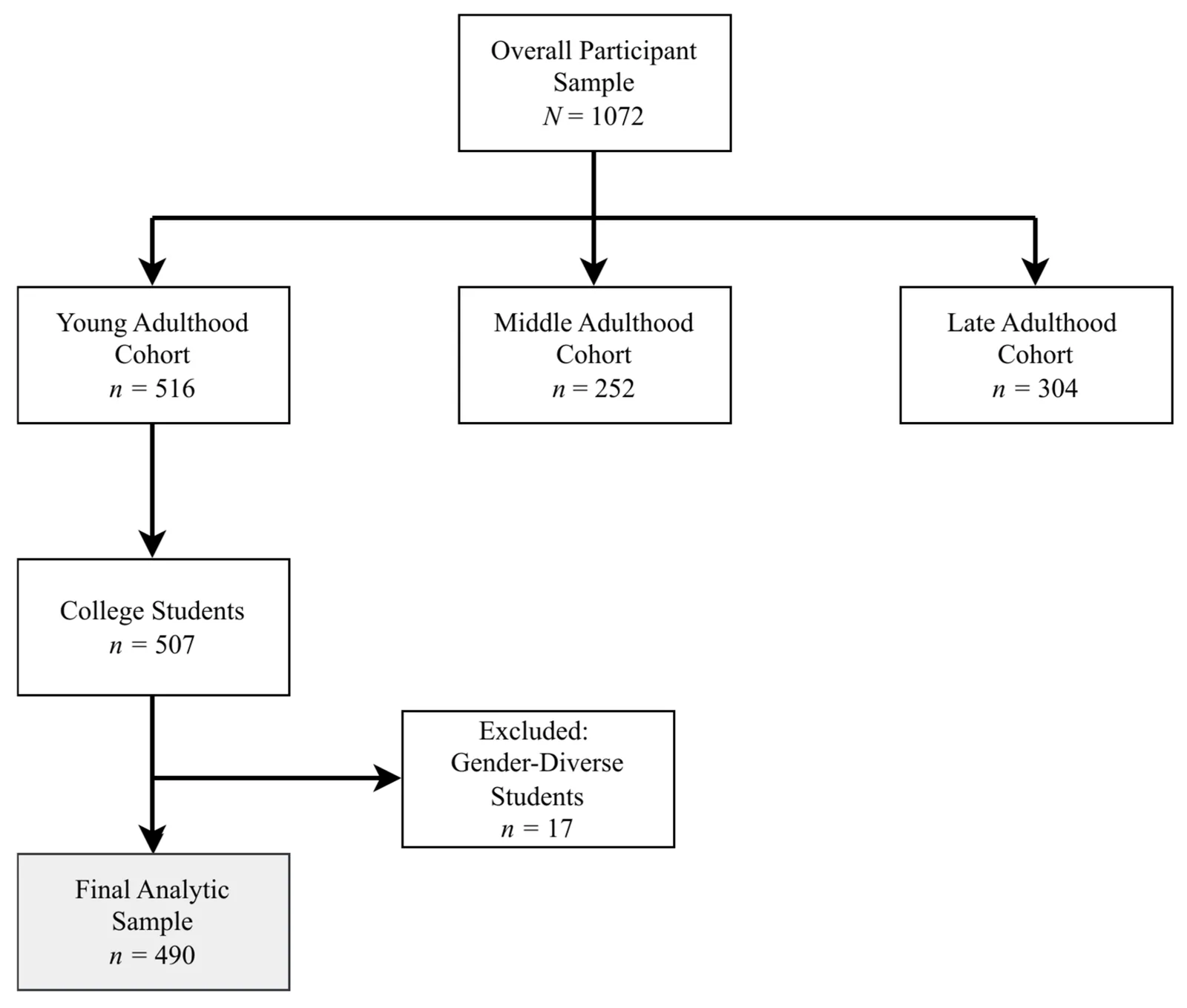
Consort diagram of overall study sample.

The second moderated mediation model assessed whether Latinx college students’ social support moderated the relations between COVID-19 experiences and COVID-19 racial/ethnic bias predicting mental health outcomes via global sleep quality. This model had acceptable fit: χ^2^ (*df* = 14) = 61.77, *p* < 0.001; RMSEA = 0.08; CFI = 0.94, SRMR = 0.04 (Figure 2). Findings from this model indicated that there were no significant moderation effects of social support. However, consistent with the other model, global sleep quality mediated the association between COVID-19 experiences and mental health, (95% CI = 0.40, 0.75) and between COVID-19 racial/ethnic bias and mental health (95% CI = 0.05, 0.31). In addition, a few direct effects were also significant such that more COVID-19 experiences were associated with worse mental health, and more social support was associated with better mental health (see Figure 3 and Table 4 for direct effect statistics). Regarding the covariate, female participants were more likely to report more severe mental health symptoms than males.

## 3. Discussion

Many stressors and disruptions to daily life and health were caused by the COVID-19 pandemic, especially for minoritized individuals. Latinx college students, for example, reported interruptions to their education and increases in general stress and worry regarding the well-being of their families and themselves [8]. In addition, there was a rise in discrimination and racial/ethnic bias during the pandemic due to anti-immigrant sentiments [9]. The present study contributes to the literature by using original survey data to explore whether global sleep quality mediated the association between COVID-19 factors (i.e., negative COVID-19 experiences and COVID-19 racial/ethnic bias) and mental health symptoms in a sample of Latinx college students. We were also interested in exploring whether cultural factors moderated this mediational model.

We found that more negative COVID-19 experiences (e.g., more exposure to the virus and more impacts to daily life) were associated with worse global sleep quality, and, in turn, more symptoms of depression and anxiety. Given that mental health functioning is one of the main causes of poor academic outcomes and possible college completion for students [41,42], the current study highlights the importance of sleep for mental health functioning. Notably, we found that over 70% of the students in the present study reported poor global sleep quality. This is consistent with, if not slightly higher than, other studies that examined sleep quality among college students during the pandemic [17,43]. Brown and Papp [44] examined sleep quality changes prior to the onset of and during the COVID-19 pandemic for college students. They reported that initial pandemic-related changes (i.e., later wake times) in sleep patterns returned to earlier pre-pandemic patterns as the pandemic progressed and students adjusted to the new normal. While stress was initially reported to be low immediately following the onset of the pandemic, college students reported more stress as the pandemic continued [44]. Longitudinal studies exploring the association between college students’ sleep and mental health, following the onset of the pandemic, remain highly relevant.

We also found that greater COVID-19 racial/ethnic bias was indirectly associated with worse mental health outcomes, through poorer global sleep quality. It is well established that racism and discrimination are associated with poorer general health, physical health, and mental health (e.g., depression, anxiety, and psychological stress [12]). In fact, our finding corresponds with the race-based disparities in stress and sleep in context model (RDSSC; [45]) that proposes that sleep disturbances serve as a biological pathway through which discrimination stress changes developmental outcomes. The effects of perceived racial/ethnic bias on the health outcomes of minoritized college students, like Latinxs, remain of significant importance, as various forms of racism and discrimination continue to be reported across colleges [46,47]. Thus, this study provides additional support for the deleterious behavioral health consequences of racial or ethnic bias on Latinx college students’ sleep health and, ultimately, their mental health.

Despite prior work demonstrating that discrimination associated with the COVID-19 pandemic was associated with increased mental health symptoms [24,25], we did not find this direct association. It is possible that sample and measurement differences between this study and prior work contribute to these mixed findings. For instance, Shi et al.’s [24] sample included adults across the lifespan, not just college students, and Yip et al. [25] measured COVID-19 racial/ethnic victimization, not bias. Furthermore, our study collected data over a longer period (10/2020 through 2/2022), in comparison to Yip et al. [25] and Shi et al. [24] whose participants rated COVID-19-related discrimination in April 2020 and October 2020, respectively. It is also possible that such an association does not exist. Overall, our findings suggest that global sleep quality plays an important role in the association between COVID-19 stressors and Latinx college students’ mental health, highlighting a potential target for stress-reducing interventions.

A strength of the current study was the focus on culturally relevant moderators. Contrary to our hypotheses, we did not find support for attitudinal *familismo* support as a moderator of the association between COVID-19 factors and mental health symptoms via global sleep quality. Stein and others [48] also did not find support of *familismo* as a buffer between the association of COVID-19 stressors and depressive and anxiety symptoms among Latinx adolescents. Further, contrary to our hypothesis, attitudinal *familismo* support was not significantly associated with mental health outcomes. Given that *familismo* is a multi-faceted cultural value that can be attitudinal or behavioral in nature, it is possible that they interact in ways that would affect direct associations. Nicasio et al. [36] recommend the evaluation of components of *familismo* as they found that attitudinal and behavioral *familismo* interact to predict depressive symptoms among Latinx adults. Within the context of COVID-19, Latinx college students may have experienced moderate levels of attitudinal *familismo* support and low levels of behavioral *familismo* support due to barriers imposed by the pandemic, thereby limiting the possible role of *familismo* on experiences associated with the pandemic and their mental health outcomes.

Further, the analyses did not support our hypotheses of social support as buffering the negative impact of COVID-19 factors on mental health outcomes through global sleep quality. Other studies have found similar results; namely, that perceived social support did not moderate the association between COVID-19 stressors and stress, anxiety, and depressive symptoms [14,49]. However, we did find that greater perceived social support was associated with better mental health outcomes, which is consistent with prior literature [38,39].

### 3.1. Constraints on Generality

This study is not without limitations. First, this is a nonrandom or representative sample of Latinx college students in the United States, so findings do not generalize to Latinx young adults, generally, or to those not in college or of different ages, specifically. It is important to note that the literature reviewed in this manuscript was primarily focused on college students in the US given the demographic characteristics of the study sample. Findings from studies across the world, including studies in Central and South America and those from the Global Chrono Corona Survey, have also found that the pandemic and lockdown impacted adult and young adults sleep and mental health [3,50]. Indeed, a study focused on the impact of the lockdown on Argentinian adults’ sleep found that the lockdown had a greater impact on young (compared to older) adults’ sleep and chronotype [51].

The findings from this study are also limited in that they do not apply to gender diverse Latinx college students. The overall study population was composed of 507 students inclusive of 10 students who identified as non-binary, 5 as genderfluid, and 2 as transgender. We chose not to analyze the data from the 17 students because we did not want to aggregate participants who identified as a gender identity other than female and male into a “non-binary” group. Thus, future studies interested in assessing for gender identity differences as they relate to significant life stressors, sleep, and mental health outcomes must carefully develop and implement recruitment strategies [52] such as oversampling gender diverse students, thereby allowing appropriate representativeness and statistical power. Given the nonrandom, predominantly female, English-speaking, largely single-university sample, findings should not be generalized to all Latinx college students without caution. Additionally, future research examining disparities in Latinx young adults’ mental and sleep health based on their country of residence’s response to the COVID-19 pandemic will be of benefit.

Another limitation worth noting involves the range of time in which participants were able to participate. Participants were invited to complete the survey approximately seven months following the start (13 March 2020) of the COVID-19 lockdown in the United States. The average time at which participants completed the survey was 13.1 months (over a year) since the start of the lockdown. As such, participants’ COVID-19 experiences and related psychosocial distress could have been influenced by changes that occurred in their access to COVID-19 prevention such as vaccination willingness [53] or attitudes [54], or their education, like class modality (i.e., virtual vs. in-person [55]). The time since the onset of the COVID-19 lockdown and the time at which the participant completed the survey was calculated and assessed as a possible covariate. Months since the onset of the pandemic was not significantly associated with the COVID-19 experiences, social support, attitudinal *familismo* support, and mental health outcomes variables. With more time that followed the onset of the pandemic, participants reported less COVID-19 racial/ethnic bias.

Additionally, this study was cross-sectional. As such, causal inferences cannot be drawn from the findings. Furthermore, the mediation analyses conducted in this study identified global sleep quality as a mediator of the mental health outcomes of Latinx college students. The cross-sectional nature of this study merits caution and identifies the need for future study of sleep quality as a mediator through longitudinal approaches to establish temporal precedence. Examining possible bidirectional associations between mental health outcomes and global sleep quality is also warranted. A few measurement limitations should also be considered. First, global sleep quality was measured through the participant’s self-report via the Pittsburgh Sleep Quality Index (PSQI; [40]). While the PSQI has been widely used to assess global sleep quality through assessment of different sleep characteristics/components (i.e., subjective sleep quality, latency, duration, efficiency, disturbances, use of sleep medications, and daytime dysfunction), other measurement techniques may have offered more nuanced measurement of sleep quality. For instance, use of actigraphy, a non-invasive method of capturing body movements while the individual sleeps, can offer objective information about sleep–wake patterns [56]. Second, degree of physical activity and quantity of light exposure were not measured and it is recommended future studies consider these variables as covariates. Finally, the structural validity of the composite measure of COVID-19 negative experiences has not been evaluated. Future studies may consider utilizing a measure that examines COVID-19 negative impacts, with similar response scales to strengthen construct validity of the measurement.

Relatedly, the survey did not assess whether participants had ever contracted the COVID-19 virus or the number of times they contracted it, nor did it explore whether they had experienced long-COVID symptoms [57]. A few studies have found that long-COVID symptoms are associated with poorer physical, sleep, and mental health outcomes [58,59]. Lastly, participants were not asked about their living circumstances or housing-related changes and stability since the onset of the pandemic. As such, it could be possible that their proximity to family or loved ones could have also influenced their access to social support. Taken together, prior contractions of COVID-19 and associated long-COVID symptoms, housing status and stability, and proximity to relatives should be considered as cofounding variables in future studies assessing COVID-19-related changes in sleep and mental health.

### 3.2. Implications

Findings from this study emphasize the importance of sleep as Latinx college students navigated pandemic stressors. The role of global sleep quality in explaining the paths between COVID-19 stressors and mental health, highlight the need to focus on sleep-related interventions to promote improved sleep and mental wellbeing. In a review, Chellappa and Aeschbach [60] identified nonpharmacological interventions, such as lifestyle changes (e.g., reducing smoking, caffeine, alcohol, and drug use) and cognitive behavioral therapy for insomnia (CBT-I) as ways of enhancing sleep in individuals experiencing anxiety. Similarly, Baroni et al. [61] also found gains in overall sleep characteristics (i.e., sleep hygiene, perceived sleep latency, and circadian sleep phase) and reductions in symptoms of depression and anxiety for college students who completed a sleep course. Thus, enhancing access and implementation of cognitive behavioral therapies at the college and university level offers an effective method of reducing depression and anxiety [62] for this vulnerable population of young adults.

Although we did not find evidence for social support or attitudinal *familismo* support as significant buffers for the negative impact of COVID 19 factors and the mental health outcomes of Latinx college students, we did find a direct effect of social support on mental health symptoms. As such, it is important to ensure that Latinx college students are aware of supportive resources available to manage psychosocial distress. To promote the mental wellbeing of Latinx college students, colleges may consider creating opportunities to bridge students with other students, networks, or organizations, and also find ways to bring their families into their programs as a way of honoring and affirming their cultural values. Seeking alternative buffers, such as perceived institutional support or resiliency building methods, may inform pathways toward Latinx college students mental health during significant periods of duress such as the pandemic or continued pandemic changes.

## 4. Materials and Methods

### 4.1. Participants

Data for this study is drawn from a larger study (*N* = 1072) conducted during the COVID-19 pandemic to examine mental health outcomes and cultural factors in three age groups (i.e., young adulthood 18–25; middle adulthood 30–50; late adulthood 55+) of Latinx individuals. The current study included data from a subset (507 Latinx college students) of the larger sample who completed measures following the start of the COVID-19 lockdown on 13 March 2020 [63]. On average, participants completed the online survey (questionnaires available in Supplementary Materials) 13.2 months (*SD* = 5.6) following lockdown, with a range of 7 to 22 months.

Table 5 presents demographic characteristics of the sample. Students’ mean age was 19.6 years (*SD* = 1.6 years) with a range from 18 to 25. Most students (70.3%) identified as female, 26.2% identified as male, and 3.3% identified as another gender identity (i.e., transgender, genderfluid, or non-binary). Given the low numbers of participants that reported a gender identity other than female or male (e.g., gender diverse), 17 cases were removed from the current study, resulting in an analytic sample size of 490 (72.9% identified as female; see Figure 3). Participants were given the option to select all that apply for their racial and ethnic background. Most students (75.7%) listed Latinx alone and 24.3% marked Latinx with a racial category or Multiracial and Latinx. Most of the students (61.4%) were freshman, 18.4% sophomores, 12.2% juniors, 6.1% seniors, and 1.8% were in graduate school. With respect to generational status, 10.6% were first generation, 53.7% were second generation immigrants, 35.7% were third generation or beyond (for definitions of immigrant generations, see Farley & Alba [64]).

### 4.2. Procedures

Eligibility criteria for the current analytic sample involved being between 18 to 25 years of age, self-identifying as Latinx/Hispanic, having the ability to read and comprehend English, and providing informed consent. Participant recruitment began in October 2020 after Institutional Review Board approval was obtained and ended February 2022. The survey was conducted online, and two recruitment methods were used: advertising the study to students from a large mid-Atlantic public university who would be eligible to receive credit for participation through the online SONA platform, and virtual flyers posted on social media platforms (e.g., Twitter, Instagram, and Facebook). Subsequent snowball sampling occurred on the virtual platforms. Interested participants could access the survey from a link on the flyer. Most of the sample (95%) was recruited through university classes for SONA credit, and the rest through public recruitment.

Participants completed multiple questionnaires compiled into a single survey. The survey was only available in English and took approximately 45 min to complete. Participants were able to complete the survey on a mobile device or computer. Student participants received research credit for study completion if enrolled through the SONA System; otherwise, participants did not receive compensation for survey completion. Participants were unable to re-enroll in the study after earning research credits or if they did not meet inclusion criteria during their first attempt.

### 4.3. Measures

#### 4.3.1. COVID-19 Experiences

The 25-item CAIR-Pandemic Impact Questionnaire (C-PIQ; [65]) includes three subscales. The current study used the COVID-19 exposure and impact subscales to create a single negative COVID-19 experience item [21]. COVID-19 exposure was assessed through 14 items (e.g., “struggled with responsibilities at home due to the coronavirus pandemic”). Participants were asked if a negative consequence of the pandemic (e.g., increased risk of virus due to job) had happened to them and someone close to them and rated each item with a yes (1) or no (0) response. One item assessing whether someone close to the participant had died of complications of the coronavirus was scored as a 2 if the participant said ‘yes’ [65]. A total exposure score was calculated by summing the self and other exposure responses marked as “yes,” with greater scores indicating greater exposure to COVID-19.

For the COVID-19 impact subscale, participants responded to six statements assessing the impact of COVID-19 on their life. Item responses varied as some assessed frequency from 0 (never) to 4 (most of the time) (e.g., “how much do you worry about your health or the health of your friends and family”), intensity from not at all (0) to extremely (4) (e.g., “how stressful have changes in social [family and friends] contacts been for you”), and duration of sleep from no loss of sleep (0) to >3 h less of sleep. An item probing sleep duration and an item about mental health were removed prior to scoring and a single negative COVID-19 experiences score was calculated by summing the exposure and impact scales consistent with prior research [21]. Higher scores indicate more negative COVID-19 experience. Cronbach’s alpha was 0.78.

#### 4.3.2. COVID-19 Racial/Ethnic Bias

The 9-item Coronavirus Racial Bias Scale (CRBS; [66]) assessed participants’ beliefs about how COVID-19 had increased negative racial/ethnic public attitudes and experiences towards them. Participants endorsed their level of agreement from 0 (strongly disagree) to 3 (strongly agree) with each statement (e.g., “I believe the country has become more dangerous for people in my racial/ethnic group because of fear of the Coronavirus”). For analyses, a summed score was used, with higher scores indicating greater COVID-19 racial/ethnic bias. Cronbach’s alpha was 0.82.

#### 4.3.3. Global Sleep Quality

The 19-item Pittsburgh Sleep Quality Index (PSQI; [40]) was used to assess participant’s global sleep quality within the past 30 days. The PSQI measures various sleep characteristics (grouped into seven components) including sleep quality, latency, duration, efficiency, disturbances, use of sleep medications, and daytime dysfunction. Item response options for each component ranged from 0 (no difficulty or low frequency) to 3 (severe difficulty or high frequency). Items were summed to create a global sleep quality score, and higher scores indicated worse global sleep quality. A cutoff score of 5 was reflective of poor global sleep quality [40]. Cronbach’s alpha was 0.70.

#### 4.3.4. Social Support

Participants completed the 12-item Multidimensional Scale of Perceived Social Support (MSPSS; [67]) measure that assesses perceived support from family, friends, and significant others. Participants rated each item from 0 (very strongly disagree) to 6 (very strongly agree) to indicate their agreement with a statement (e.g., “my family really tries to help me” or “I can count on my friends when things go wrong”). Items were summed with higher scores indicating greater social support. Cronbach’s alpha was 0.92.

#### 4.3.5. Attitudinal Familismo Support

The Mexican American Cultural Values Scale (MACVS; [68]) assesses Latinx cultural values. The current study utilized the attitudinal *familismo* support subscale. Participants responded to six items that asked about their values relating to support given to and received by family. Items (e.g., “Family provides a sense of security because they will always be there for you”) were rated on a 5-point Likert scale ranging from 0 (not at all) to 4 (completely). Items were summed with higher scores indicating greater attitudinal *familismo* support. Cronbach’s alpha was 0.90.

#### 4.3.6. Depressive Symptoms

Participants completed the 9-item Patient Health Questionnaire-9 (PHQ-9; [69]). Participants rated each item on a scale from 0 (not at all) to 3 (nearly every day) to indicate how frequently in the past two weeks they were bothered by a particular problem (e.g., “little interest or pleasure in doing things”). A single item assessing sleep as it relates to depressive symptoms was removed prior to scoring for path analyses. To estimate symptom severity, all nine items were summed (range 0 to 27), with higher scores indicating greater depression symptom severity. Scores of 5, 10, 15, and 20 represent cutoff scores for mild, moderate, moderately severe, and severe depression, respectively [69]. Reliability for this measure was α = 0.89.

#### 4.3.7. Anxiety Symptoms

The 7-item Generalized Anxiety Disorder Scale-7 (GAD-7; [70]) was used to assess participant’s symptoms of anxiety within the past two weeks. Participants rated each item on a scale from 0 (not at all) to 3 (nearly every day) to indicate how often they experienced various problems (e.g., “worrying too much about different things”). Items were summed, with higher scores indicating greater symptom severity (range 0 to 21). Scores of 5, 10, 15, and ≥15 represent cutoff scores for minimal, mild, moderate, and severe anxiety, respectively [70]. Cronbach’s alpha was 0.92.

### 4.4. Analytic Approach

First, we verified that there were no missing values. The construction of the survey required that participants respond to all items; thus, there were no missing data across all variables. Second, a latent variable was created, which involved the loadings of manifest variables (i.e., depressive symptoms and anxiety symptoms) onto the mental health latent variable. A mediation model was tested using IBM SPSS version 29 AMOS through Structural Equation Modeling to assess whether global sleep quality mediated the association between COVID-19 variables and mental health symptoms. We tested significant mediation by examining the product of coefficients of the mediating effect with bias-corrected bootstrapped (2000 replications) confidence intervals. Any confidence intervals excluding 0 indicated significant mediation [71,72]. We tested the moderating role of attitudinal *familismo* support and social support in the pathway from COVID-19 variables predicting mental health via global sleep quality. For these moderated mediation models, we ran one model that included attitudinal *familismo* support and one model that included social support. Significant interaction effects were assessed through simple slope analyses, as recommended by Preacher et al. [73], and were graphed at one standard below or above the mean of the moderating variables. To assess how well each of the models fit the data, we examined fit indices, including the comparative fit index (CFI), the root-mean-square error of approximation (RMSEA), and the standardized root-mean-square residual (SRMR). For the CFI, values above 0.90 indicate adequate fit and above 0.95 indicate good fit [74]. An RMSEA below 0.10 and SRMR below 0.08 indicates acceptable fit, and below 0.05 indicates good fit [74]. Gender identity was included as a covariate in all models. Given prior research has documented differences in the mental health outcomes of college students based on their age [75] and gender identity [76,77,78], these two variables were considered as possible covariates. Immigrant generation and racial/ethnic identity were also evaluated as potential covariates as they may contribute to the mental health outcomes of Latinx young adults [79,80]. In consideration of the range of time in which participants completed the assessment battery, a variable assessing time since the start of the COVID-19 pandemic was also evaluated as a potential covariate. Covariate selection was evaluated through bivariate correlations prior to formal path analyses to reduce possible overfitting and increase reliability.

## 5. Conclusions

The current study contributes to the literature by highlighting the important role of sleep during times of stress. Findings indicate that poorer global sleep quality explained the association between more negative COVID-19 experiences (e.g., more exposure to the COVID-19 virus and impacts to daily life due to the pandemic) and more perceived COVID-19 racial/ethnic bias with worsened mental health outcomes. This study also examined whether perceived attitudinal *familismo* support and social support buffered the association between COVID-19 factors and mental health outcomes. While there were no significant interaction effects observed, there was still evidence of a direct association between perceived social support and mental health outcomes.

## Figures and Tables

**Table 1:** Correlations, means, and standard deviations among study variables.

Variable	*M*	*SD*	Range	1.	2.	3.	4.	5.	6.	7.
1. Gender Identity	72.9%	0.4		-						
2. COVID-19 Experiences	14.8	5.5	0–31	0.20 ***	-					
3. COVID-19 Racial/Ethnic Bias	11.2	5.3	0–27	0.10 *	0.26 ***	-				
4. Social Support	52.0	13.5	0–72	0.19 ***	0.04	−0.03	-			
5. *Familismo*	16.6	5.0	0–24	0.03	0.04	−0.03	0.31 ***	-		
6. Global Sleep Quality	8.0	3.8	0–21	0.08	0.35 ***	0.18 ***	−0.15 **	−0.12 *	-	
7. Mental Health Outcomes	-	-	-	0.16 **	0.42 ***	0.22 ***	−0.20 ***	−0.12 *	0.48 ***	-

Note. * *p* < 0.05, ** *p* < 0.01, *** *p* < 0.001; attitudinal *familismo* support = *familismo*. Positive values for global sleep quality indicate poorer global sleep quality and positive values for mental health outcomes indicate more severe mental health symptoms of anxiety and depression.

**Table 2:** Mediation analysis summary.

Association	β	*SE*	*p*	Confidence Interval	
				LL	UL
Direct Effects					
COVID-19 Experiences → Global Sleep Quality	0.32	0.04	0.001 **	0.25	0.39
COVID-19 Racial/Ethnic Bias → Global Sleep Quality	0.10	0.04	0.028 *	0.03	0.17
COVID-19 Experiences → Mental Health Outcomes	0.27	0.05	0.001 **	0.18	0.35
COVID-19 Racial/Ethnic Bias → Mental Health Outcomes	0.08	0.05	0.06	0.01	0.17
Global Sleep Quality → Mental Health Outcomes	0.37	0.05	0.001 **	0.28	0.44
Gender → Mental Health Outcomes	0.09	0.04	0.05	0.01	0.15
Indirect Effects					
COVID-19 Experiences → Global Sleep Quality → Mental Health Outcomes	0.12	0.02	0.001 **	0.09	0.16
COVID-19 Racial/Ethnic Bias → Global Sleep Quality → Mental Health Outcomes	0.04	0.02	0.023 *	0.01	0.07
Total Effects					
COVID-19 Experiences → Global Sleep Quality → Mental Health Outcomes	0.39	0.05	0.002 **	0.29	0.46
COVID-19 Racial/Ethnic Bias → Global Sleep Quality → Mental Health Outcomes	0.12	0.05	0.002 **	0.05	0.20

Note. * *p* < 0.05, ** *p* < 0.01; lower limit = LL, upper limit = UL. Positive values for global sleep quality indicate poorer global sleep quality and positive values for mental health outcomes indicate more severe mental health symptoms of anxiety and depression.

**Table 3:** Moderated mediation analysis summary—attitudinal *familismo* support.

Association	β	*SE*	*p*	Confidence Interval	
				LL	UL
Direct Effects					
COVID-19 Experiences → Global Sleep Quality	0.32	0.04	<0.001 ***	0.25	0.39
COVID-19 Racial/Ethnic Bias → Global Sleep Quality	0.10	0.04	0.025 *	0.03	0.18
COVID-19 Experiences → Mental Health Outcomes	0.27	0.05	<0.001 ***	0.19	0.36
COVID-19 Racial/Ethnic Bias → Mental Health Outcomes	0.08	0.04	0.08	0.00	0.15
Global Sleep Quality → Mental Health Outcomes	0.36	0.05	<0.001 ***	0.28	0.44
*Familismo* → Mental Health Outcomes	−0.08	0.05	0.09	−0.16	−0.00
COVID-19 Experiences × *Familismo* → Mental Health Outcomes	0.04	0.05	0.47	−0.04	0.12
COVID-19 Racial/Ethnic Bias × *Familismo* → Mental Health Outcomes	0.09	0.05	0.09	0.01	0.17
Gender → Mental Health Outcomes	0.09	0.04	0.037 *	0.02	0.16
Indirect Effects					
COVID-19 Experiences → Global Sleep Quality → Mental Health Outcomes	0.12	0.02	<0.001 ***	0.09	0.16
COVID-19 Racial/Ethnic Bias → Global Sleep Quality → Mental Health Outcomes	0.04	0.02	0.031 *	0.01	0.07
Total Effects					
COVID-19 Experiences → Global Sleep Quality → Mental Health Outcomes	0.37	0.05	<0.001 ***	0.30	0.45
COVID-19 Racial/Ethnic Bias → Global Sleep Quality → Mental Health Outcomes	0.12	0.05	0.010 *	0.04	0.19

Note. * *p* < 0.05, *** *p* < 0.001; lower limit = LL, upper limit = UL; attitudinal *familismo* support = *familismo*. Positive values for global sleep quality indicate poorer global sleep quality and positive values for mental health outcomes indicate more severe mental health symptoms of anxiety and depression.

**Table 4:** Moderated mediation analysis summary—social support.

Association	β	*SE*	*p*	Confidence Interval	
				LL	UL
Direct Effects					
COVID-19 Experiences → Global Sleep Quality	0.33	0.04	<0.001 ***	0.25	0.39
COVID-19 Racial/Ethnic Bias → Global Sleep Quality	0.10	0.04	0.025 *	0.03	0.17
COVID-19 Experiences → Mental Health Outcomes	0.26	0.05	<0.001 ***	0.18	0.35
COVID-19 Racial/Ethnic Bias → Mental Health Outcomes	0.08	0.02	0.07	0.01	0.15
Global Sleep Quality → Mental Health Outcomes	0.35	0.05	<0.001 ***	0.27	0.42
Social Support → Mental Health Outcomes	−0.17	0.05	<0.001 ***	−0.26	−0.10
COVID-19 Experiences × Social Support → Mental Health Outcomes	−0.04	0.05	0.41	−0.11	0.05
COVID-19 Racial/Ethnic Bias × Social Support → Mental Health Outcomes	0.06	0.05	0.24	−0.02	0.15
Gender → Mental Health Outcomes	0.11	0.04	0.010 *	0.04	0.18
Indirect Effects					
COVID-19 Experiences → Global Sleep Quality → Mental Health Outcomes	0.11	0.02	<0.001 ***	0.08	0.15
COVID-19 Racial/Ethnic Bias → Global Sleep Quality → Mental Health Outcomes	0.03	0.02	0.031 *	0.01	0.06
Total Effects					
COVID-19 Experiences → Global Sleep Quality → Mental Health Outcomes	0.39	0.05	<0.001 ***	0.30	0.46
COVID-19 Racial/Ethnic Bias → Global Sleep Quality → Mental Health Outcomes	0.11	0.05	0.011 *	0.04	0.19

Note. * *p* < 0.05, *** *p* < 0.001; LL = lower limit, UL = upper limit; positive values for global sleep quality indicate poorer global sleep quality and positive values for mental health outcomes indicate more severe mental health symptoms of anxiety and depression.

**Table 5:** Demographic characteristics of the sample (*n* = 490).

Demographic Variable	*n* (%)
Age (years), mean (*SD*)	19.1 (1.6)
Gender Identity	
Male	133 (27.1)
Female	357 (72.9)
Race/Ethnicity	
Latinx	371 (75.7)
Black/African American and Latinx	45 (9.2)
White and Latinx	44 (9.0)
Asian and Latinx	12 (2.4)
American Indian or Alaska Native and Latinx	2 (0.4)
Middle Eastern or North African and Latinx	2 (0.4)
Native Hawaiian or Other Pacific Islander and Latinx	1 (0.2)
Multiracial and Latinx	13 (2.7)
Academic Year	
Freshman	301 (61.4)
Sophomore	90 (18.4)
Junior	60 (12.2)
Senior	30 (6.1)
Graduate Student	9 (1.8)
Country of Origin	
United States or U.S. Territory	440 (89.8)
Mexican	6 (1.2)
Central America	22 (4.5)
South America	16 (3.3)
Other	6 (1.2)
Immigrant Generation	
First Generation	52 (10.6)
Second Generation	263 (53.7)
Third Generation or Beyond	175 (35.7)
Family Socioeconomic Class	
Poor or low-income	48 (9.8)
Working class	151 (30.8)
Middle class	279 (56.9)
Rich or upper-class	12 (2.4)

Note. U.S. territory refers to Puerto Rico.

## Data Availability

The data that support the findings of this study are available from the corresponding author upon reasonable request to researchers who provide the appropriate signed data use agreement.

## Supplementary Materials

The following supporting information can be downloaded at: https://www.mdpi.com/article/10.3390/clockssleep8030049/s1, Questionnaire information including: Demographics, CAIR Pandemic Impact Questionnaire (C-PIQ), Coronavirus Racial Bias Scale (CRBS), Pittsburgh Sleep Quality Index (PSQI), Multidimensional Scale of Perceived Social Support (MSPSS), Mexican American Cultural Values Scale (MACVS), Patient Health Questionnaire-9 (PHQ-9) [81], and Generalized Anxiety Disorder-7 (GAD-7).
