# Supplementary material for: The Impact of the COVID-19 Pandemic on Latinx College Students’ Mental Health: Exploring the Role of Sleep, Social Support, and Attitudinal Familismo Support
Source: Clocks Sleep. 2026 Aug 21;8(3):49. doi: 10.3390/clockssleep8030049 (PMC13511539; doi:10.3390/clockssleep8030049)
Supplement: Supplementary file 1 [file clockssleep-08-00049-s001.zip › clockssleep-4423594-supplementary.pdf]

Demographics

1. What is your age in years?
2. What age group do you belong to?
    a. 25 to 34 years old
    b. 35 to 44 years old
    c. 45 to 54 years old
    d. 55 to 64 years old
    e. 65 to 74 years old
    f. 75 years or older
3. Which term do you use describe your gender identity?
    a. Male
    b. Female
    c. Transgender
    d. Agender
    e. Genderfluid
    f. Non-binary
    g. My identity is not listed above (please specify)
4. What socioeconomic class would you classify your family in?
    a. Poor or low-income
    b. Working class
    c. Middle class
    d. Rich or upper-class
5. What is your current classification as a student?
    a. Freshman
    b. Sophomore
    c. Junior
    d. Senior
    e. Other
6. Describe your current marital status
    a. Single
    b. Married
    c. Separated
    d. Divorced
    e. Widowed
    f. Other
7. Are you currently dating or in a relationship
    a. Yes
    b. No
8. What is your country of origin?
    a. United States
    b. Other
9. How old were you (in years and or months) when you moved to the USA to live
10. What language was spoken at home when you were a child?
    a. Only English
    b. Only Spanish

    c. Both English and Spanish
    d. Other language (please specify)
11. What is your father's country of origin?
    a. United States
    b. Other
12. Is your father deceased
    a. Yes
    b. No
13. If yes, please select the age range that you were when he passed?
    a. 0-5 years (infancy through the preschool years)
    b. 6-12 years (school aged)
    c. 13-17 (adolescent)
    d. 18-25 (emerging adulthood)
    e. 26 years and older (adulthood)
14. What is your mother's country of origin?
    a. United States
    b. Other
15. Is your mother deceased
    a. Yes
    b. No
16. If yes, please select the age range that you were when she passed?
    a. 0-5 years (infancy through the preschool years)
    b. 6-12 years (school aged)
    c. 13-17 (adolescent)
    d. 18-25 (emerging adulthood)
    e. 26 years and older (adulthood)

CAIR Pandemic Impact Questionnaire (C-PIQ)

Complementary and Integrative Research (CAIR) Lab, PI Ariel J Lang, PhD, MPH (ajlang@health.ucsd.edu).

Measure adapted from MacLean and Cloitre, 2020 (CRISIS V0.2) and Taku, Cann, Calhoun and Tedeschi, 2008.

For more information about the measure, please refer to the following source: Lang A. J. (2020). CAIR Pandemic Impact Questionnaire (C-PIQ). National Institute of Environmental Health Sciences. https://tools.niehs.nih.gov/dr2/files/cairpiq_scoring_508.pdf

The Coronavirus Racial Bias Scale (CRBS)

Fordham University Pathways to Health Study

Copyright 2020 Fisher, Celia B. & Yip, Tiffany. Fordham University Center for Ethics Education.

For more information about the measure, please refer to the following source: Fisher, C.B., & Yip, T. (2020) The Coronavirus Racial Bias Scale (CRBS). Fordham University. https://www.fordham.edu/Coronavirus_Racial_Bias_Scale_Fordham_CRBS.pdf

Pittsburgh Sleep Quality Index (PSQI)

Copyright 1989 and 2010. University of Pittsburgh. All rights reserved. Developed by Buysse, D.J., Reynolds, C.F., Monk, T.H., Berman, S.R., and Kupfer, D.J. of the University of Pittsburgh using National Institute of Mental Health Funding.

For more information about accessing the instrument, please see the following link: https://www.sleep.pitt.edu/psqi

Buysse DJ, Reynolds CF, Monk TH, Berman SR, Kupfer DJ: Psychiatry Research, 28:193-213, 1989. Contact Mapi Research Trust for information on translated versions at: https://eprovide.mapi-trust.org/

Multidimensional Scale of Perceived Social Support (MSPSS)

The MSPSS is in the public domain and free to use for clinical and research purposes.

Zimet, G. D., Dahlem, N. W., Zimet, S. G., & Farley, G. K. (1988). The multidimensional scale of perceived social support. *Journal of Personality Assessment*, *52*(1), 30–41. https://doi.org/10.1207/s15327752jpa5201_2

1. There is a special person who is around when I am in need.
    a. Very Strongly Disagree
    b. Strongly Disagree
    c. Mildly Disagree
    d. Neutral
    e. Mildly Agree
    f. Strongly Agree
    g. Very Strongly Agree
2. There is a special person with whom I can share my joys and sorrows.
    a. Very Strongly Disagree
    b. Strongly Disagree
    c. Mildly Disagree
    d. Neutral
    e. Mildly Agree
    f. Strongly Agree
    g. Very Strongly Agree
3. My family really tries to help me.
    a. Very Strongly Disagree
    b. Strongly Disagree
    c. Mildly Disagree
    d. Neutral
    e. Mildly Agree
    f. Strongly Agree
    g. Very Strongly Agree
4. I get the emotional help and support I need from my family.
    a. Very Strongly Disagree
    b. Strongly Disagree
    c. Mildly Disagree
    d. Neutral
    e. Mildly Agree
    f. Strongly Agree
    g. Very Strongly Agree
5. I have a special person who is a real source of comfort to me.
    a. Very Strongly Disagree

    b. Strongly Disagree
    c. Mildly Disagree
    d. Neutral
    e. Mildly Agree
    f. Strongly Agree
    g. Very Strongly Agree
6. My friends really try to help me.
    a. Very Strongly Disagree
    b. Strongly Disagree
    c. Mildly Disagree
    d. Neutral
    e. Mildly Agree
    f. Strongly Agree
    g. Very Strongly Agree
7. I can count on my friends when things go wrong.
    a. Very Strongly Disagree
    b. Strongly Disagree
    c. Mildly Disagree
    d. Neutral
    e. Mildly Agree
    f. Strongly Agree
    g. Very Strongly Agree
8. I can talk about my problems with my family.
    a. Very Strongly Disagree
    b. Strongly Disagree
    c. Mildly Disagree
    d. Neutral
    e. Mildly Agree
    f. Strongly Agree
    g. Very Strongly Agree
9. I have friends with whom I can share my joys and sorrows.
    a. Very Strongly Disagree
    b. Strongly Disagree
    c. Mildly Disagree
    d. Neutral
    e. Mildly Agree
    f. Strongly Agree
    g. Very Strongly Agree
10. There is a special person in my life who cares about my feelings.
    a. Very Strongly Disagree

b. Strongly Disagree
c. Mildly Disagree
d. Neutral
e. Mildly Agree
f. Strongly Agree
g. Very Strongly Agree

11. My family is willing to help me make decisions.
    a. Very Strongly Disagree
    b. Strongly Disagree
    c. Mildly Disagree
    d. Neutral
    e. Mildly Agree
    f. Strongly Agree
    g. Very Strongly Agree
12. I can talk about my problems with my friends.
    a. Very Strongly Disagree
    b. Strongly Disagree
    c. Mildly Disagree
    d. Neutral
    e. Mildly Agree
    f. Strongly Agree
    g. Very Strongly Agree

Mexican American Cultural Values Scale (MACVS)

Test content may be reproduced and used for non-commercial research and educational purposes. Distribution must be controlled, meaning only to the participants engaged in the research or enrolled in the educational activity.

Knight, George P., Gonzales, Nancy A., Saenz, Delia S., Bonds, Darya D., Germán, Miguelina, Deardorff, Julianna, Roosav, Mark W., & Updegraff, Kimberly A. (2010). The Mexican American Cultural Values Scale for adolescents and adults. The Journal of Early Adolescence, Vol 30(3), 444-4481. https://dx.doi.org/10.1177/0272431609338178, © 2010 by SAGE Publications.

Patient Health Questionnaire – 9 (PHQ-9)

Test content is public domain.

Spitzer, Robert L. (1999). Validation and utility of a self-report version of PRIME-MD: The PHQ Primary Care Study. JAMA: Journal of the American Medical Association, 282(18), 1737-1744. doi: 10.1001/jama.282.18.1737

Over the last 2 weeks, how often have you been bothered by any of the following problems?

1. Little interest or pleasure in doing things.
    a. Not at all
    b. Several days
    c. More than half the days
    d. Nearly every day
2. Feeling down, depressed, or hopeless.
    a. Not at all
    b. Several days
    c. More than half the days
    d. Nearly every day
3. Trouble falling asleep or staying asleep, or sleeping too much.
    a. Not at all
    b. Several days
    c. More than half the days
    d. Nearly every day
4. Feeling tired or having little energy.
    a. Not at all
    b. Several days
    c. More than half the days
    d. Nearly every day
5. Poor appetite or overeating.
    a. Not at all
    b. Several days
    c. More than half the days
    d. Nearly every day
6. Feeling bad about yourself – or that you are a failure or have let yourself or your family down.
    a. Not at all
    b. Several days
    c. More than half the days
    d. Nearly every day
7. Trouble concentrating on things, such as reading the newspaper or watching television.

    a. Not at all
    b. Several days
    c. More than half the days
    d. Nearly every day
8. Moving or speaking so slowly that other people could have noticed. Or the opposite – being so fidgety or restless that you have been moving around a lot more than usual.
    a. Not at all
    b. Several days
    c. More than half the days
    d. Nearly every day
9. Thoughts that you would be better off dead or of hurting yourself.
    a. Not at all
    b. Several days
    c. More than half the days
    d. Nearly every day

Generalized Anxiety Disorder – 7 (GAD-7)

Test content is public domain.

Spitzer, R. L., Kroenke, K., Williams, J. B., & Löwe, B. (2006). A brief measure for assessing generalized anxiety disorder: the GAD-7. Archives of Internal Medicine, 166(10), 1092–1097. https://doi.org/10.1001/archinte.166.10.1092

Over the last 2 weeks, how often have you been bothered by any of the following problems?

1. Feeling nervous, anxious or on edge
    a. Not at all
    b. Several days
    c. More than half the days
    d. Nearly every day
2. Not being able to stop or control worrying
    a. Not at all
    b. Several days
    c. More than half the days
    d. Nearly every day
3. Worrying too much about different things
    a. Not at all
    b. Several days
    c. More than half the days
    d. Nearly every day
4. Trouble relaxing
    a. Not at all
    b. Several days
    c. More than half the days
    d. Nearly every day
5. Being so restless that it is hard to sit still
    a. Not at all
    b. Several days
    c. More than half the days
    d. Nearly every day
6. Becoming easily annoyed or irritable
    a. Not at all
    b. Several days
    c. More than half the days
    d. Nearly every day
7. Feeling afraid as if something awful might happen
    a. Not at all

b. Several days
c. More than half the days
d. Nearly every day
